# Staged antibiotic-loaded cement-based reconstruction versus flap-based reconstruction for complex diabetic foot defects in patients with osteoporosis: a retrospective cohort study

**DOI:** 10.3389/fsurg.2026.1834390

**Published:** 2026-05-20

**Authors:** Hanlin Zhang, Desheng Chen, Qiang Song, Fan Gong, Peiling Li, Yu Li, Le Fei, Yanchuan Yang, Anyang Yu

**Affiliations:** 1 Department of Orthopedics Center, People’s Hospital of Ningxia Hui Autonomous Region, Ningxia Medical University, Yinchuan, Ningxia, China; 2 The Third Clinical Medical College, Ningxia Medical University, Yinchuan, Ningxia, China; 3 General Practice Department, People’s Hospital of Ningxia Hui Autonomous Region, Ningxia Medical University, Yinchuan, Ningxia, China

**Keywords:** AB-NPWT, antibiotic-loaded bone cement, diabetic foot, diabetic foot osteomyelitis, flap transplantation, limb salvage, osteoporosis, retrospective cohort study

## Abstract

**Objective:**

This retrospective cohort study aimed to compare the clinical efficacy and safety of a staged antibiotic-loaded bone cement combined with negative pressure wound therapy (AB-NPWT) pathway vs. flap transplantation in the treatment of chronic foot ulcers with soft tissue defects in patients with type 2 diabetes mellitus (T2DM) and osteoporosis.

**Methods:**

We retrospectively analyzed 56 patients with T2DM and osteoporosis who underwent surgical treatment between January 2020 and December 2024. Treatment allocation was non-randomized and determined by the attending surgeon; no formal sample size calculation was performed. In the AB-NPWT group, 5 patients (17.2%) crossed over to receive local flap coverage as part of the staged pathway. The analysis was not adjusted for multiple comparisons. The AB-NPWT group (*n* = 29) and flap transplantation group (*n* = 27) were compared for perioperative parameters, wound healing, limb salvage, complications, and AOFAS scores. Adjusted analyses (multivariable regression and propensity score matching) were performed to account for potential confounding.

**Results:**

Operative time and blood loss were significantly lower in the AB-NPWT group (both *p* < 0.001). At 12 months, complete wound healing (86.2% vs. 88.9%, *p* = 0.754), limb salvage (93.1% vs. 92.6%, *p* = 1.000), and median healing time (126 vs. 112 days, *p* = 0.221) did not differ significantly between groups. Complication rates were similar (24.1% vs. 33.3%, *p* = 0.446), but profiles differed. AOFAS scores at final follow-up were comparable (72.5 ± 10.3 vs. 75.8 ± 11.6, *p* = 0.270).

**Conclusions:**

In this retrospective exploratory cohort, no statistically significant between-group differences were detected in the main clinical outcomes. AB-NPWT was associated with reduced surgical trauma, whereas flap transplantation provided definitive coverage in a single operative session after debridement. Treatment selection should be individualized based on wound characteristics, patient systemic status, and healthcare resources. These findings are hypothesis-generating and require confirmation in larger prospective trials.

## Introduction

The coexistence of type 2 diabetes mellitus (T2DM) and osteoporosis (OP) represents a clinically challenging comorbidity that exacerbates the pathological processes of each condition. Patients with T2DM have a 20%–40% higher risk of hip fracture compared to non-diabetic individuals, with increased post-fracture mortality (1–4). When such patients develop chronic diabetic foot ulcers, the dual metabolic–mechanical defect—impaired microcirculation and neuropathy from diabetes combined with compromised skeletal integrity from osteoporosis—creates a vicious cycle: ulcers rapidly progress to osteomyelitis with extensive soft tissue defects, leading to prolonged healing, doubled amputation risk, and elevated mortality (5, 6).

Contemporary standards of diabetic foot care, as outlined in the 2023 International Working Group on the Diabetic Foot (IWGDF) and Infectious Diseases Society of America (IDSA) guidelines on diabetes-related foot infections, emphasize that infection management must not be considered in isolation but rather integrated with pressure off-loading, peripheral vascular assessment and revascularization when indicated, and metabolic control (7, 8). Similarly, the 2023 IWGDF guidelines on interventions to enhance healing of foot ulcers stress that advanced wound-healing technologies should be used as adjuncts to established multidisciplinary care, not as stand-alone treatments (9). The 2023 IWGDF practical guidelines further synthesize these principles into a comprehensive multidisciplinary framework for prevention and management of diabetes-related foot disease (10).

Despite the growing recognition of this high-risk population, evidence-based surgical strategies tailored to T2DM–OP patients remain scarce. Two aggressive surgical reconstruction approaches are commonly employed. Antibiotic-loaded bone cement combined with negative pressure wound therapy (AB-NPWT) provides local infection control and dead space management, achieving limb salvage rates of 75%–97% in diabetic foot osteomyelitis (11). Conversely, flap transplantation—especially free flaps—offers definitive, well-vascularized coverage, with reported success rates exceeding 90% in complex foot defects (12, 13). A sequential approach combining antibiotic cement with flap reconstruction has shown promising short-term outcomes in diabetic calcaneal osteomyelitis (14).

However, current evidence has two critical limitations. First, most studies treat “diabetic foot” as a homogeneous entity, overlooking the distinct impact of osteoporosis on surgical outcomes. Bone quality directly influences cement anchorage and flap fixation on osteoporotic bone surfaces, yet this subgroup has not been specifically analyzed (15). Second, no high-level evidence directly compares AB-NPWT and flap transplantation in T2DM–OP patients. Existing literature consists mainly of single-arm studies or comparisons with conservative management, leaving surgeons without a clear framework for choosing between these resource-intensive strategies.

To address this gap, we conducted a retrospective cohort study directly comparing AB-NPWT and flap transplantation—both with standardized negative pressure therapy—in T2DM–OP patients presenting with chronic foot ulcers complicated by osteomyelitis and soft tissue defects. By evaluating perioperative parameters, wound healing, complications, limb salvage, and functional recovery, we aimed to answer: For this specific high-risk population, which strategy offers a superior risk–benefit profile? Comparative data specifically in this high-risk subgroup remain limited. This study provides preliminary comparative observational data to inform future research and, potentially, clinical decision-making within the limitations of a retrospective design.

## Methods

### Study design and setting

This was a single-center, retrospective cohort study conducted at the Department of Orthopedics Center, People's Hospital of Ningxia Hui Autonomous Region, between January 2020 and December 2024. The study protocol was approved by the Institutional Ethics Committee (Approval No. 2026-LL-088). The requirement for informed consent was waived due to the retrospective use of anonymized data.

### Study population

**Figure 1:**
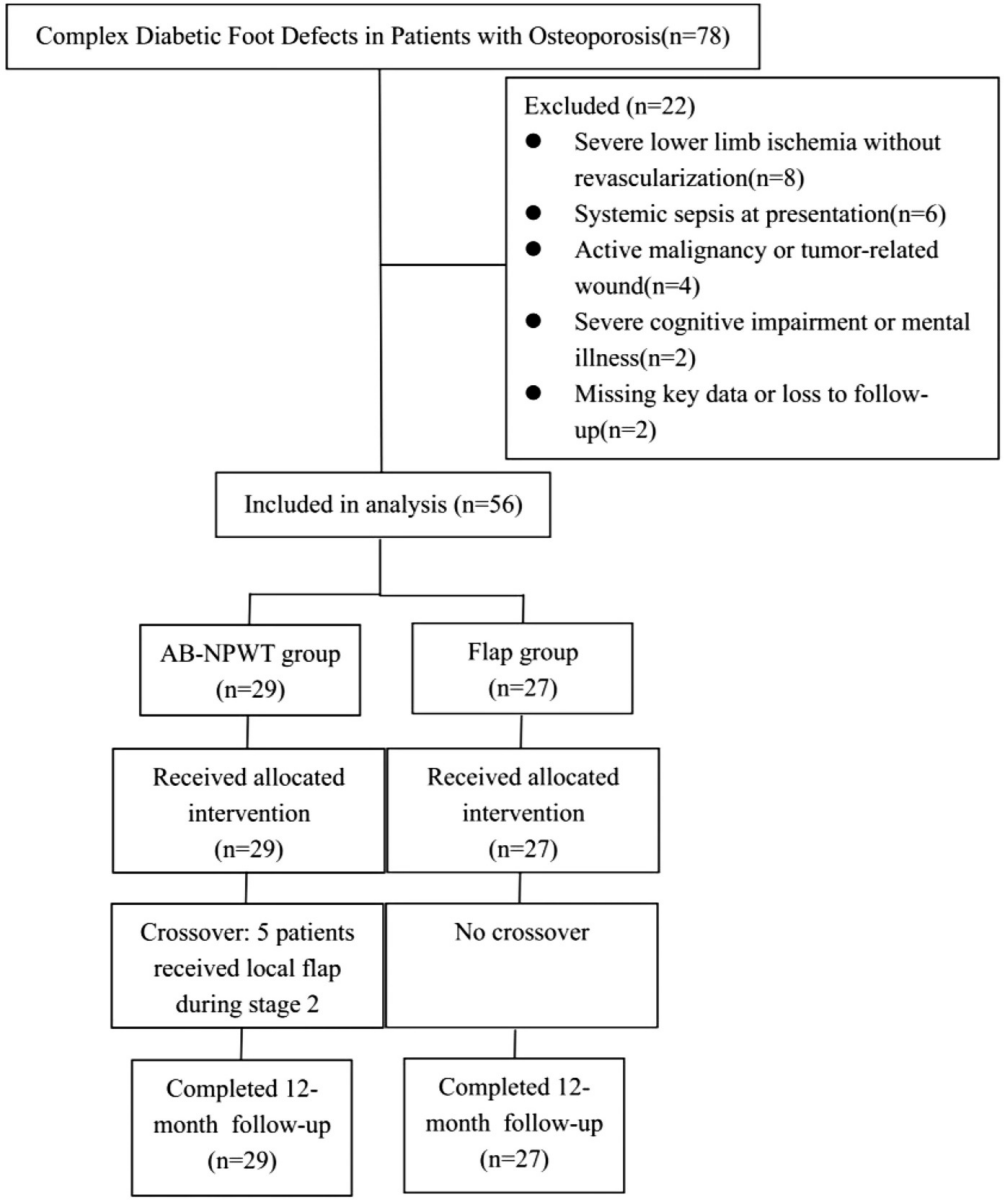
Participant flow diagram. A total of 78 patients with type 2 diabetes mellitus and osteoporosis were screened. Twenty-two patients were excluded for the reasons shown. The remaining 56 patients were included in the final analysis, with 29 allocated to the AB-NPWT pathway and 27 to the flap-based pathway. In the AB-NPWT group, 5 patients (17.2%) crossed over to receive local flap coverage during Stage 2 reconstruction. All 56 patients completed 12-month follow-up.

We retrospectively screened all patients with T2DM and osteoporosis who underwent surgical treatment for chronic foot ulcers (Wagner grade 3 or 4) with confirmed osteomyelitis and soft tissue defects. Inclusion criteria were: (1) age ≥18 years; (2) T2DM duration >5 years; (3) osteoporosis diagnosed by dual-energy x-ray absorptiometry (T-score ≤ –2.5 at lumbar spine or hip) according to WHO criteria (16) or Chinese guidelines (17); (4) chronic ulcer (>4 weeks) with osteomyelitis confirmed by imaging (x-ray, MRI) and/or pathology; (5) soft tissue defect requiring surgical coverage; (6) first-time receipt of either target surgical procedure. Exclusion criteria were: (1) severe lower limb ischemia (ankle–brachial index <0.5 or toe–brachial index <0.3) without successful revascularization; (2) systemic sepsis; (3) active malignancy or tumor-related wound; (4) severe cognitive impairment or mental illness; (5) missing key data or loss to follow-up. A total of 78 patients screened, 22 were excluded for reasons detailed in Figure 1. The remaining 56 patients met all inclusion criteria and were included in the final analysis. Among the 56 patients included in the final analysis, there were no missing data for any baseline covariate or outcome variable used in the primary or secondary analyses. Consequently, no imputation methods were applied.

### Group allocation

**Figure 2:**
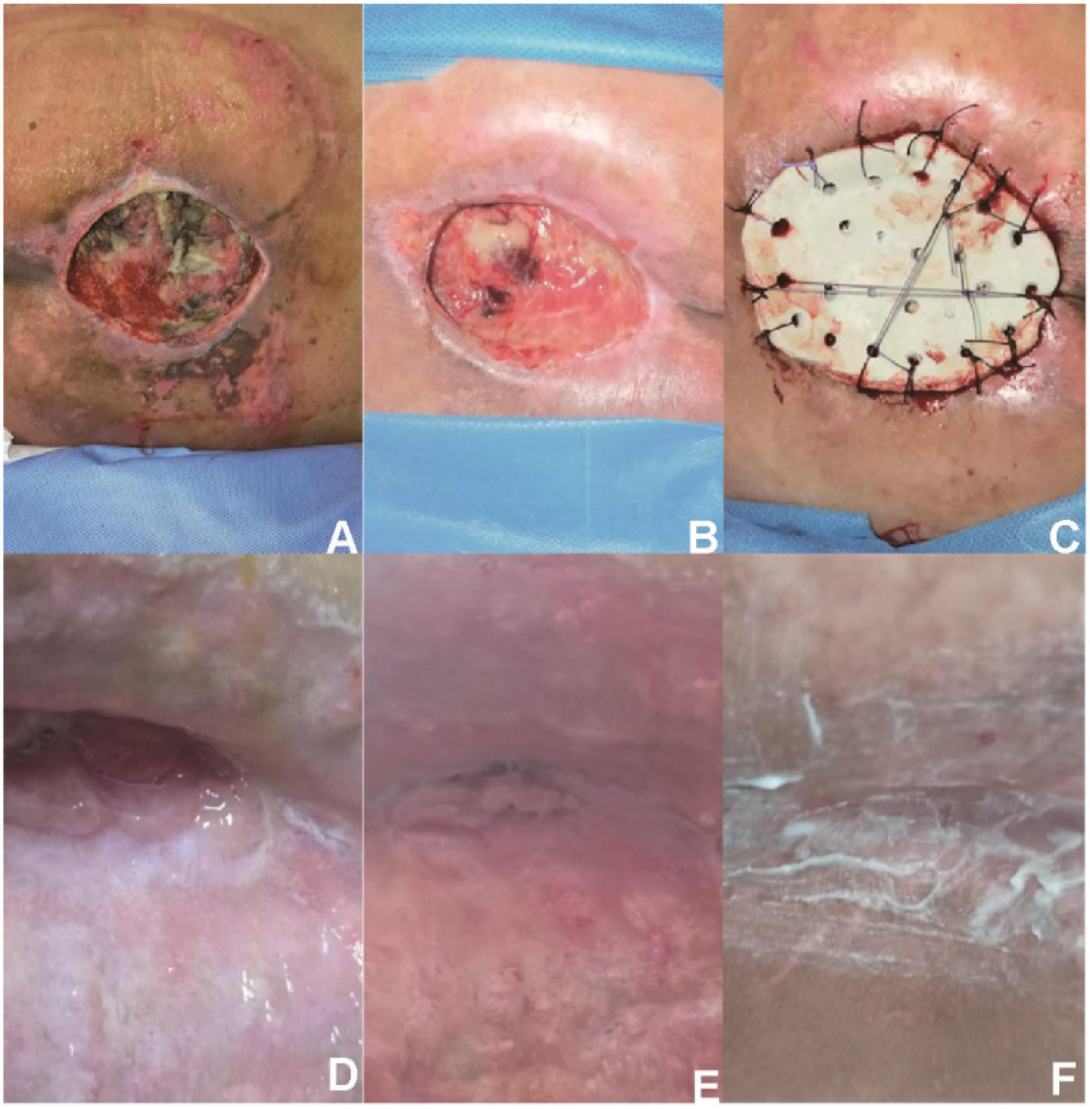
A 68-year-old female with type 2 diabetes mellitus and osteoporosis presented with a chronic complex foot defect. **(A)** Preoperative view showing a soft tissue defect with exposed bone and necrotic tissue. **(B)** Wound bed after radical debridement and one week of negative pressure wound therapy (NPWT), demonstrating clean granulation tissue without purulence. **(C)** Placement of antibiotic-loaded cement spacer (Palacos® R + G with vancomycin 2 g per 40 g cement) into the defect, covered with NPWT foam. **(D)** Five months after cement removal and split-thickness skin grafting, showing early re-epithelialization. **(E)** Seven months postoperatively, progressive wound contraction and healing. **(F)** Nine months postoperatively, complete wound closure with stable scar formation. No flap procedure was performed in this patient.

Patients were allocated to two reconstructive pathways based on the definitive coverage strategy performed after initial debridement and infection control:

AB-NPWT-based pathway (*n* = 29): Patients first received antibiotic-loaded bone cement (polymethylmethacrylate mixed with vancomycin, 2 g per 40 g cement) to fill bone and soft tissue defects, followed by NPWT. After granulation tissue formation, secondary wound closure was achieved using split-thickness skin grafting (*n* = 19, 65.5%), direct closure (*n* = 5, 17.2%), or small local flaps (*n* = 5, 17.2%). Thus, the AB-NPWT pathway is a staged approach in which cement serves as the primary infection-control and dead-space-management tool, with subsequent coverage individualized (Figure 2).

**Figure 3:**
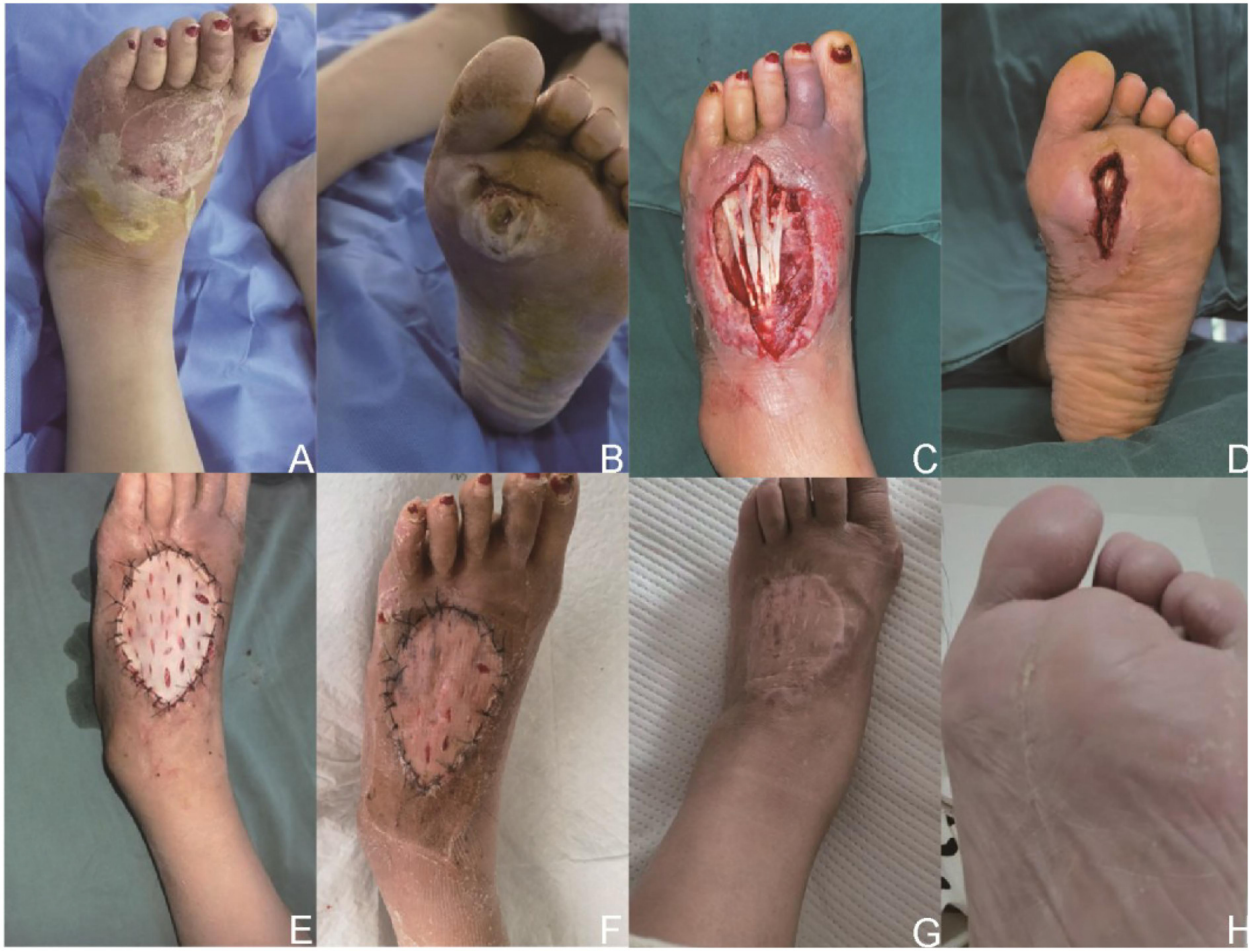
A 65-year-old male with type 2 diabetes mellitus and osteoporosis presented with a chronic complex foot defect. **(A,B)** Preoperative views showing plantar ulceration with exposed calcaneus and surrounding soft tissue loss. **(C,D)** Wound condition after Stage 1 debridement and negative pressure wound therapy (NPWT), revealing a clean, well-vascularized wound bed. **(E)** Immediate postoperative appearance after free anterolateral thigh flap transplantation and split-thickness skin grafting. **(F)** Two weeks after flap surgery, showing early graft integration and flap viability. **(G,H)** Eight weeks postoperatively, demonstrating stable flap survival, complete wound healing, and restored foot integrity.

Flap-based pathway (*n* = 27): Patients underwent flap transplantation as the definitive coverage procedure after debridement and NPWT. Flap types included local flaps (*n* = 11, 40.7%), pedicled flaps (*n* = 9, 33.3%), and free flaps (*n* = 7, 25.9%). All flap patients also received postoperative NPWT (Figure 3).

The choice of pathway was determined by the attending surgeon based on wound characteristics, patient condition, and shared decision-making; therefore, allocation was non-randomized and reflects real-world clinical practice. The comparison is therefore between two reconstructive pathways rather than between two discrete interventions.

### Treatment selection algorithm

The choice between the AB-NPWT-based and flap-based reconstructive pathways was made by the attending surgeon based on a structured clinical assessment. The following factors were systematically considered:

1. Exposed bone or tendon: Presence of critical structures (e.g., calcaneus, Achilles tendon) without periosteum or paratenon favored the flap pathway to provide well-vascularized coverage. When these structures were covered by granulation tissue or could be protected by cement, the AB-NPWT pathway was considered.
2. Defect size and location: Large soft tissue defects (>10 cm^2^) or defects involving weight-bearing surfaces (plantar heel, metatarsal heads) typically prompted flap pathway. Smaller defects (<10 cm^2^) or non-weight-bearing areas (dorsal foot) were managed with AB-NPWT.
3. Infection burden: Active purulent infection, residual necrotic bone after debridement, or microbiologically confirmed osteomyelitis with multi-drug resistant organisms favored AB-NPWT for local antibiotic elution. When infection was controlled after Stage 1 debridement, either pathway was feasible.
4. Perfusion status: Patients with peripheral arterial disease (ABI < 0.6 or TcPO₂ < 30 mmHg) were not candidates for flap pathway unless successful revascularization was performed first. After revascularization, those with improved perfusion (ABI > 0.7) could undergo flap pathway; those with persistently marginal perfusion received AB-NPWT.
5. Donor-site feasibility: Flap pathway required adequate donor tissue (local skin laxity for local flaps; suitable perforators for pedicled/free flaps). Patients with prior flap harvest, extensive lower limb trauma, or contraindications to prolonged surgery were directed to AB-NPWT.
6. Systemic frailty and comorbidities: High surgical risk (ASA physical status ≥3, severe COPD, ejection fraction <35%, advanced CKD stage 4–5, or liver cirrhosis) favored the less invasive AB-NPWT pathway. Fit patients with good functional reserve were candidates for flap pathway.
7. Microsurgical expertise and resources: Free flap reconstruction was performed only when a dedicated microsurgery team and intraoperative monitoring (Doppler, near-infrared spectroscopy) were available. In the absence of such resources, local flaps or AB-NPWT were selected.
8. Patient preference: After informed discussion of risks, benefits, number of procedures, and recovery expectations, patient preference was respected.

**Figure 4:**
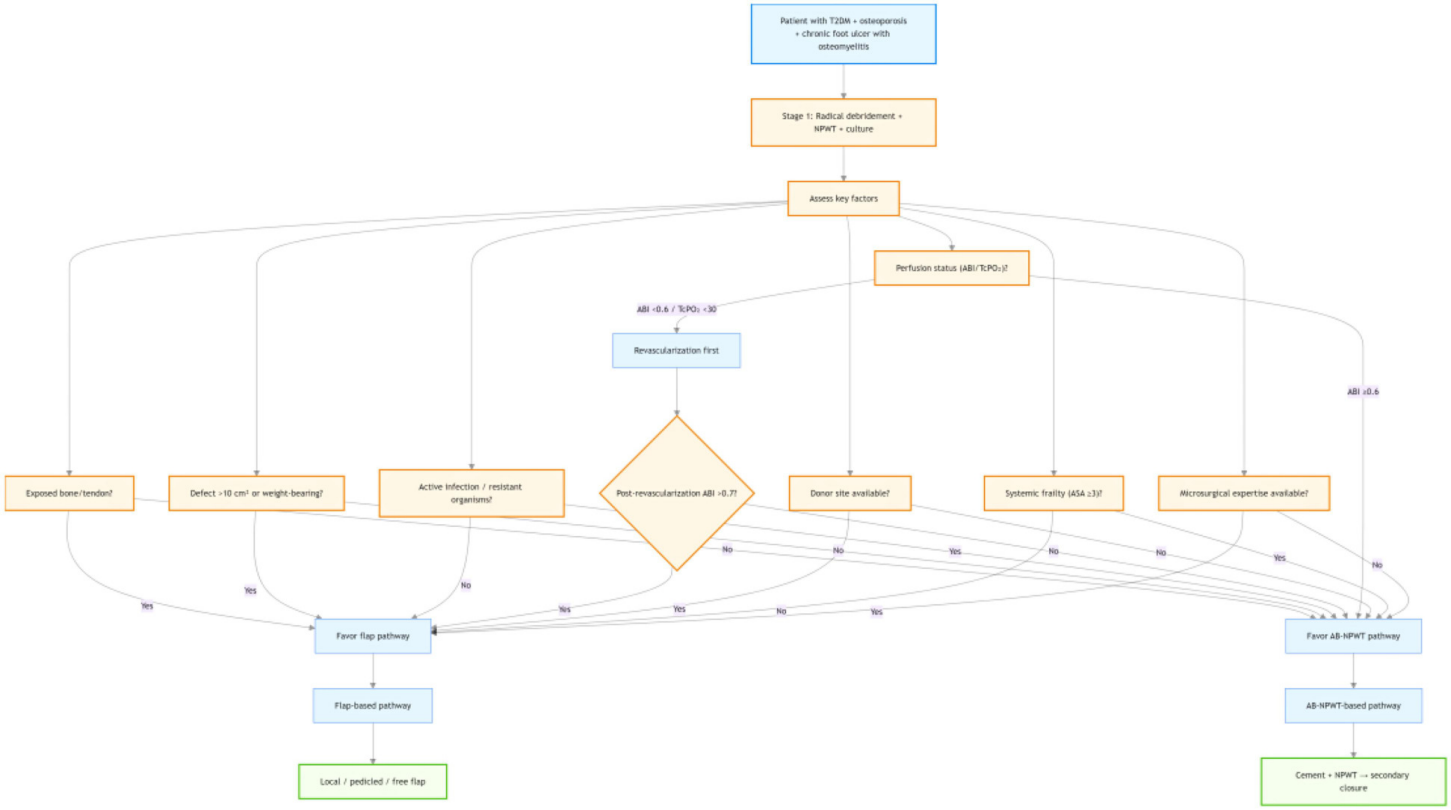
Conceptual diagram of the treatment selection algorithm used to assign patients to either the AB-NPWT-based or flap-based reconstructive pathway. Decisions were based on clinical assessment of exposed bone/tendon, defect size and location, infection burden, perfusion status, donor-site feasibility, systemic frailty, and availability of microsurgical expertise. The algorithm was applied after Stage 1 debridement and NPWT, once infection was controlled.

The final decision was made collaboratively by the attending surgeon (senior author with >10 years of experience in diabetic foot reconstruction) and the patient. This algorithm was applied consistently throughout the study period. A conceptual diagram of the decision process is provided in Figure 4.

### Treatment procedures

All patients received multidisciplinary management including glycemic control (target fasting glucose <10 mmol/L), vascular assessment, nutritional support, and anti-osteoporosis treatment (calcium 1,000 mg/day, vitamin D 800 IU/day, and bisphosphonates as prescribed). Surgical treatment followed a standardized two-stage approach:

**Stage 1–Radical debridement and NPWT:** All patients underwent radical debridement with pulse lavage, followed by NPWT at −125 mmHg, changed every 48–72 h. Targeted intravenous antibiotics were administered for at least 2 weeks based on culture results, followed by oral antibiotics for a total course of 6–12 weeks.

**Criteria for Proceeding to Stage 2:** The decision to proceed from Stage 1 (debridement and NPWT) to Stage 2 (definitive reconstruction) was based on a structured assessment of four domains. All four criteria had to be met before surgery was scheduled. The assessments were performed weekly after Stage 1.

- Wound appearance (clinical domain): All of the following had to be absent: purulent drainage, periwound erythema (>1 cm), local warmth, and malodor. In addition, the wound bed had to show healthy granulation tissue covering ≥75% of the defect area (assessed by the attending surgeon using a sterile ruler).
- Inflammatory markers (laboratory domain): Serum C-reactive protein (CRP) and white blood cell count (WBC) were measured weekly. Criteria for infection control were: (a) a decreasing trend over two consecutive measurements (≥30% reduction from peak or from previous week), and (b) CRP <10 mg/L (normal reference: <5 mg/L; we used <10 mg/L as acceptable for diabetic patients with chronic inflammation) and WBC <10 × 10⁹/L. If a patient had an elevated baseline CRP due to another condition (e.g., rheumatoid arthritis, active malignancy), the criterion was a return to that patient's individual baseline.
- Microbiological findings (culture domain): At the time of Stage 1 debridement, multiple deep tissue samples (minimum 2) were sent for aerobic and anaerobic culture. Before proceeding to Stage 2, a repeat deep tissue culture was performed at the bedside or in the operating room during a “second-look” procedure. Criteria for infection control were: (a) no growth of the original pathogen(s) on repeat culture, or (b) if cultures remained positive, the organism had to be of low virulence (e.g., coagulase-negative Staphylococcus) and the patient had received at least 2 weeks of targeted antibiotics with clinical improvement. Superficial swabs were never used for this decision.
- Imaging (radiological domain, when indicated): MRI was repeated if there was clinical suspicion of residual osteomyelitis (e.g., persistent focal bone pain, sinus tract, failure of inflammatory markers to normalize). Criteria for proceeding were: resolution of bone marrow edema (T2 STIR sequence) and absence of new or worsening abscess formation. Plain radiographs were obtained in all patients at week 4 to assess for progressive bone destruction, but were not used as standalone criteria.

Scoring system and timing: We used a simplified Infection Control Score (ICS) where each of the four domains was scored as 0 (not controlled) or 1 (controlled). A score of 4 (all domains controlled) was required to proceed. The median time to achieve infection control was 5 weeks (IQR 4–7 weeks). Patients who did not meet criteria by week 8 underwent repeat debridement and another NPWT cycle. No patient in this cohort required more than two cycles before Stage 2. The final decision was documented by the attending surgeon in the patient' s medical record. This protocol was applied uniformly to both treatment pathways.

**Stage 2–Definitive coverage:** All procedures were performed by or under the direct supervision of the senior author (D.C., with >15 years of experience in diabetic foot reconstruction).

**AB-NPWT pathway (cement-based reconstruction)**—The following protocol was used:

- Cement product: Palacos® R + G (Heraeus Medical, Germany), a pre-mixed polymethylmethacrylate cement containing gentamicin (0.5 g per 40 g powder). An additional 2 g of vancomycin powder (Hospira, USA) was manually mixed per 40 g cement to achieve a final vancomycin concentration of approximately 5% w/w.
- Spacer configuration: For cavitary defects (<30 cm^3^), cement was rolled into 5–8 mm beads and strung on a 2-0 absorbable polyglactin suture (Vicryl®, Ethicon) to form a “cement chain”. For larger defects, a custom-molded block spacer was created using a sterile syringe barrel as a mold. Beads or spacers were placed loosely into the defect without tension.
- Protection of critical structures: Exposed tendons, nerves, and vessels were first covered with a layer of absorbable collagen membrane (Terudermis®, Olympus Terumo Biomaterials) to prevent direct cement abrasion. The cement spacer was then placed, followed by a single layer of non-adherent dressing (Mepitel®, Mölnlycke) before NPWT application.
- Temporary vs. definitive: The cement spacer was always intended as a temporary device. It was left *in situ* for 4–6 weeks to allow granulation tissue formation and local antibiotic elution.
- Removal protocol: At 4–6 weeks, a second-look procedure was performed. If the wound showed healthy granulation tissue (>75% coverage, no purulence, negative deep culture), the cement beads/spacer were removed piecemeal using a curette and rongeur. The resulting cavity was then closed secondarily (direct closure, skin graft, or small local flap as described). If infection persisted, the cement spacer was replaced with a new vancomycin-loaded cement spacer, and NPWT was reapplied for another 2–4 weeks.
- NPWT after cement: NPWT was applied at −125 mmHg continuous pressure, with foam changed every 48–72 h.

**Flap-based pathway—The following protocol was used:**

- Flap types and selection:

Local flaps (40.7% of flap group): Rotation, advancement, or transposition flaps raised from adjacent skin. Used for small to moderate defects (<5 cm diameter) with adequate surrounding skin laxity.

Pedicled flaps (33.3%): Reverse sural artery flap (for heel or Achilles defects), medial plantar flap (for plantar forefoot), or peroneal artery flap (for lateral ankle). Flaps were rotated through a subcutaneous tunnel without microvascular anastomosis.

Free flaps (25.9%): Anterolateral thigh flap (primary choice, *n* = 5), latissimus dorsi muscle flap (*n* = 1), or gracilis myocutaneous flap (*n* = 1). All free flaps were transferred to the foot defect.

- Recipient vessel strategy: For free flaps, the posterior tibial artery and its venae comitantes were used as the primary recipient vessels. End-to-side arterial anastomosis was performed to preserve distal foot perfusion; venous anastomosis was end-to-end. The dorsalis pedis artery and concomitant veins were used as an alternative when the posterior tibial vessels were unavailable or calcified.
- Postoperative monitoring protocol:
  - First 48 h: Flap color, capillary refill (<3 s), turgor, and temperature were assessed hourly by a dedicated nurse.
  - Handheld Doppler (8 MHz): Arterial signal checked hourly for 48 h, then every 2 h for the next 24 h, then every 4 h until day 7.
  - Implantable Doppler (Cook-Swartz Doppler probe): Used for free flaps (*n* = 4); the probe was placed around the arterial anastomosis and removed on day 5.
- Flap compromise and salvage protocol:
  - Venous congestion (dark color, rapid capillary refill, swelling): Bedside leech therapy (Hirudo medicinalis) if mild; if severe or persistent, emergency return to operating room for venous re-anastomosis or supercharging with a second vein.
  - Arterial insufficiency (pale, no Doppler signal, cold): Emergency exploration (<2 h from diagnosis), thrombectomy with Fogarty catheter, and revision of anastomosis. If unsuccessful, a new anastomosis was created proximal to the original site.
  - Partial necrosis (<30% of flap): Debridement of necrotic tissue and secondary intention healing or skin grafting. Total flap loss (none occurred in this series) would have prompted salvage with a second flap or conversion to AB-NPWT pathway.
- NPWT after flap: NPWT was applied over the flap at −75 mmHg (lower than for cement) to avoid compression of the microvascular anastomosis, changed every 48 h for 5–7 days.

### Diagnosis of osteomyelitis and microbiological sampling protocol

The diagnosis of diabetic foot osteomyelitis was established according to the 2023 IWGDF/IDSA guidelines, requiring at least one of the following: (a) positive bone culture, (b) positive histopathology (neutrophilic infiltration of bone), or (c) unequivocal imaging evidence (MRI with bone marrow edema, sinus tract to bone, or sequestrum) in the presence of clinical signs of infection.

#### Specimen source and timing

- Bone biopsy was the gold standard and was obtained in all patients (*n* = 56). Superficial swabs were never used for diagnosis of osteomyelitis.
- Timing: Biopsy was performed before the initiation of systemic antibiotics whenever possible (*n* = 48, 85.7%). In patients who had received antibiotics prior to referral (*n* = 8, 14.3%), antibiotics were withheld for at least 2 weeks (if clinically safe) before biopsy, or a percutaneous CT-guided biopsy was obtained through non-infected soft tissue.

#### Specimen collection technique

- Intraoperative bone biopsy: During Stage 1 debridement, after superficial debridement of granulation and necrotic soft tissue, a sterile rongeur was used to obtain a 5–10 mm^3^ piece of bone from the margin of the ulcer base (where bone was visible or palpable). Two separate bone samples were taken: one for microbiology (placed in a sterile anaerobic transport vial), one for histopathology (placed in 10% formalin).
- Percutaneous CT-guided biopsy (*n* = 4, 7.1%): For patients with no open wound or when intraoperative biopsy was not feasible, a radiologist performed a core needle biopsy (11-gauge Bonopty® needle) under CT guidance, targeting abnormal marrow on MRI. This was done through intact skin after sterile preparation.

#### Specimen processing (microbiology)

- The bone specimen was transported to the laboratory within 30 min in an anaerobic transport medium (Portagerm™, bioMérieux).
- Culture: Specimen was homogenized using a sterile mortar and pestle, then inoculated onto:
  - Blood agar (aerobic, 35 °C, 48 h)
  - Chocolate agar (5% CO_2_, 35 °C, 48 h)
  - MacConkey agar (aerobic, 35°C, 48 h)
  - Brucella blood agar with hemin and vitamin K1 (anaerobic, 35 °C, 5–7 days)
  - Thioglycollate broth (enrichment, incubated for 14 days)
- Identification: MALDI-TOF mass spectrometry (Vitek® MS, bioMérieux).
- Antibiotic susceptibility testing: Broth microdilution (Vitek® 2) according to CLSI M100 standards.
- Definitions: Osteomyelitis was confirmed if bone culture grew a pathogenic organism or histopathology showed inflammatory cells within bone trabeculae (see below). Mixed growth of ≥3 organisms was considered contamination if histopathology was negative.

#### Specimen processing (histopathology)

- Formalin-fixed, paraffin-embedded bone was decalcified (10% EDTA for 7–14 days), sectioned (5 μm), stained with hematoxylin and eosin (H&E).
- Diagnostic criteria for osteomyelitis: Presence of neutrophilic infiltrate in bone marrow or within bone trabeculae, with or without osteoclastic resorption and necrosis. Soft tissue inflammation alone (without bone involvement) was not considered osteomyelitis.
- All slides were reviewed by a senior pathologist blinded to clinical data.

#### Antibiotic therapy

- Empirical antibiotics (piperacillin-tazobactam 4.5 g q8 h or meropenem 1 g q8 h) were started immediately after Stage 1 debridement and bone biopsy, then tailored based on culture and susceptibility results.
- Targeted intravenous antibiotics were continued for a minimum of 2 weeks (range 2–6 weeks), followed by oral antibiotics (e.g., levofloxacin + clindamycin, or doxycycline + rifampicin for MRSA) to complete a total course of 6–12 weeks.
- The choice of antibiotic-loaded cement (vancomycin) was based on the most common local pathogens (Staphylococcus aureus, including MRSA).

### Multidisciplinary diabetic foot care protocol (co-interventions)

All patients received standardized multidisciplinary care according to our institutional protocol, which aligns with the 2023 IWGDF guidelines. Co-interventions were identical for both treatment pathways and were delivered by a dedicated diabetic foot team (endocrinologist, vascular surgeon, orthopaedic surgeon, podiatrist, wound care nurse, and dietitian).

#### Off-loading strategy and weight-bearing restrictions

Pre-Stage 2 (debridement and NPWT phase): Patients were strictly non-weight-bearing on the affected foot. A knee-high, posterior splint with toe-touch weight-bearing using crutches or a walker was used.

After Stage 2 (definitive coverage).

- For AB-NPWT pathway patients: After cement removal and secondary closure (skin graft, direct closure, or small local flap), patients remained non-weight-bearing for 2 weeks. Thereafter, progressive weight-bearing was allowed using a removable cast walker (RCW) with a pressure-relieving insole, starting at 25% body weight and increasing by 25% every week as tolerated.
- For flap pathway patients: Non-weight-bearing for 4 weeks post-flap to protect the microvascular anastomosis. A total contact cast (TCC) was applied for the first 2 weeks (changed weekly), followed by a RCW for the next 2 weeks. Full weight-bearing in therapeutic footwear was permitted after 4 weeks if flap healing was complete and no complications occurred.

Compliance with off-loading was monitored using a step-counter in the RCW (patients were instructed to keep steps <500 per day during non-weight-bearing phases).

#### Footwear and pressure-relief measures (post-healing)

After complete wound healing (full epithelialization), all patients received custom-molded therapeutic footwear (Diamond®, Aetrex Worldwide) with multi-density orthotic insoles to redistribute plantar pressures. Follow-up visits with a podiatrist occurred at 3, 6, and 12 months to adjust or replace footwear as needed.

#### Dressing schedule and wound care protocol

Pre-Stage 2 (during NPWT): NPWT was applied at −125 mmHg (cement pathway) or −75 mmHg (flap pathway) as described. The foam was changed every 48–72 h by a trained wound care nurse.

After Stage 2 (post-definitive coverage).

- For AB-NPWT pathway (after cement removal and secondary closure): Dry sterile gauze dressing with povidone-iodine ointment was applied daily for the first week, then every other day until complete healing. Dressings were changed by the patient or caregiver after training.
- For flap pathway (post-flap): Non-adherent dressing (Mepitel®) with saline-moistened gauze, changed daily for the first week by the wound care nurse, then every other day by the patient/caregiver. No ointments or antiseptics were applied directly to the flap.

All dressing changes were performed under aseptic technique. Signs of infection (erythema, warmth, purulence, odor) were assessed at each change.

#### Revascularisation pathway

All patients underwent preoperative vascular assessment: ankle-brachial index (ABI), toe-brachial index (TBI), and, if indicated, duplex ultrasound or CT angiography.

Indications for revascularisation: ABI < 0.5 or TBI < 0.3, or TcPO_2_ < 30 mmHg, or non-healing wound despite 4 weeks of conservative care.

Procedure: Endovascular revascularisation (balloon angioplasty ± stenting) was the first-line approach. Surgical bypass (femoral-to-pedal or popliteal-to-pedal) was reserved for patients with long-segment occlusions or failed endovascular attempts.

Timing: Revascularisation was performed before Stage 2 (definitive coverage). In the AB-NPWT group, 4 patients (13.8%) underwent endovascular revascularisation; in the flap group, 5 patients (18.5%) underwent revascularisation (4 endovascular, 1 surgical bypass).

Post-revascularisation, patients received dual antiplatelet therapy (aspirin 100 mg + clopidogrel 75 mg daily) for 1 month, then single antiplatelet indefinitely.

#### Glycaemic control

Target fasting plasma glucose: <10 mmol/L (180 mg/dL) pre-operatively and during hospitalization. HbA1c target <8.0% (but individualised for frail patients).

Management: Insulin sliding scale or basal-bolus regimen adjusted by endocrinologist. Continuous glucose monitoring was used in patients with labile diabetes (*n* = 12).

#### Nutritional support

All patients received nutritional assessment by a dietitian at baseline. Targets: serum albumin ≥35 g/L, prealbumin ≥0.2 g/L.

Supplementation: Oral nutritional supplements (Ensure®) 2 cans/day for patients with albumin <30 g/L. Enteral or parenteral nutrition was not required in this cohort.

#### Anti-osteoporosis therapy

All patients with T-score ≤ –2.5 received: calcium 1,000 mg/day, vitamin D 800 IU/day, and bisphosphonates (alendronate 70 mg weekly or zoledronic acid 5 mg annually). No patient received teriparatide or denosumab.

#### Patient education and self-care

All patients received structured education on: daily foot inspection, proper footwear, off-loading adherence, signs of infection, and glucose monitoring. Education was provided by a diabetes educator at baseline and at 1, 3, 6, and 12 months.

### Outcome measures

**Primary Outcome Measures:** Complete wound healing rate (defined as full epithelialization without need for dressings within 12 months) and limb salvage rate (avoidance of major amputation at or above the ankle).

**Secondary Outcome Measures:** Healing time (days from initial surgery to complete healing; patients who underwent amputation were censored at the time of amputation), perioperative parameters [Operative time (minutes): Stage 1, Stage 2, and cumulative (Stage 1 + Stage 2). For patients requiring additional procedures (e.g., cement removal, secondary closure), all operative episodes were summed; Intraoperative blood loss (mL): Stage 1, Stage 2, and cumulative (Stage 1 + Stage 2); Postoperative hospital stay (days): defined as total inpatient days from initial admission until discharge after the final reconstructive procedure (excluding planned readmissions for cement removal if performed as outpatient, but including any unplanned readmissions); Number of surgical procedures: total operations per patient (Stage 1 debridement + NPWT; Stage 2 definitive coverage; plus any additional procedures such as cement removal, secondary closure, or flap revision)], complication rates (infection recurrence, material/flap-related complications, systemic complications), ulcer recurrence rate, and functional status at final follow-up assessed by the AOFAS ankle-hindfoot score. Ulcer recurrence was defined as a new ulcer occurring at the same or adjacent anatomical site after complete wound healing (full epithelialization), assessed within 12 months from the date of healing. The AOFAS assessment was performed at the patient's last clinic visit, with follow-up durations ranging from 12 to 26 months. To account for variable timing, we (a) report the exact distribution of follow-up durations per group, (b) perform a sensitivity analysis restricted to patients assessed at 12 months (±1 month), and (c) use linear regression adjusting for follow-up duration as a continuous covariate.

### Confounder-adjusted analyses

To address confounding by indication, we performed both multivariable regression and propensity score-based analyses. Potential confounders were selected *a priori* based on clinical knowledge, the treatment-selection algorithm, and the literature: age, Wagner grade (3 vs. 4), ulcer area (cm^2^), peripheral arterial disease (ABI < 0.9), smoking status, baseline HbA1c, exposed bone or tendon (yes/no), and ASA physical status (I–III). The ASA score was used as a global measure of systemic frailty and comorbidity burden. Other treatment-selection variables described in the allocation algorithm (donor-site feasibility, microsurgical resource availability, patient preference) were not recorded in a standardised, quantifiable manner and therefore could not be adjusted for; this limitation is addressed in the Discussion. For binary outcomes (complete wound healing at 12 months, limb salvage), multivariable logistic regression was used, with treatment group as the independent variable and the above confounders as covariates. For healing time (censored at amputation or last follow-up), Cox proportional hazards regression was performed, reporting hazard ratios (HR) with 95% confidence intervals. Propensity scores were estimated using a logistic regression model with the same confounders. One-to-one nearest-neighbor matching without replacement was performed with a caliper of 0.2. Balance was assessed using standardized differences, with values <0.1 indicating good balance. As a sensitivity analysis, inverse probability of treatment weighting (IPTW) was also applied. All adjusted analyses were performed using SPSS version 27.0 and R version 4.2 (package “MatchIt”).

### Statistical analysis

All analyses used SPSS 27.0 and R 4.2 (“MatchIt”, “survival”, “tableone”). Continuous variables: mean ± SD (*t*-test) if normal, median (IQR, Mann–Whitney U) if non-normal. Categorical: frequencies (%) (chi-square/Fisher's). To address confounding by indication, we used eight pre-specified covariates in all multivariable and propensity score models: age, Wagner grade, ulcer area, PAD, smoking, HbA1c, exposed bone/tendon, and ASA score. Other allocation factors (donor-site feasibility, microsurgical resources, patient preference) were not standardised and could not be adjusted for (see Discussion). Binary outcomes (complete healing, limb salvage) used logistic regression → aOR (95% CI). Healing time (censored at amputation) used Cox regression → aHR (95% CI). AOFAS score used linear regression (adjusting for follow-up) → adjusted mean difference. Propensity scores were estimated with the same eight covariates; 1:1 nearest-neighbour matching (caliper 0.2) was performed, with balance assessed by standardized differences (target < 0.1). The proportional-hazards assumption was tested with Schoenfeld residuals (global *p* > 0.05 indicates no violation). A sensitivity analysis excluded the 5 crossover patients. All effect estimates are reported with 95% CI; *p*-values are two-tailed (significance at *p* < 0.05), exact to three decimals except *p* < 0.001. No adjustment for multiple comparisons due to exploratory nature.

## Results

### Baseline characteristics

A total of 78 patients were screened for eligibility. Of these, 22 were excluded for the following reasons: severe lower limb ischemia without successful revascularization (ankle-brachial index <0.5 or toe-brachial index <0.3, *n* = 8); systemic sepsis at presentation (*n* = 6); active malignancy or tumor-related wound (*n* = 4); severe cognitive impairment or mental illness (*n* = 2); and missing key data or loss to follow-up (*n* = 2). The remaining 56 patients met all inclusion criteria and were included in the final analysis (AB-NPWT group, *n* = 29; flap group, *n* = 27). A participant flow diagram is presented in Figure 1. Baseline characteristics are shown in Table 1. Standardized differences were <0.2 for all variables except diabetes duration (0.21) and BMD T-score (0.22), indicating acceptable balance between groups. The largest difference was observed for current smoking (standardized difference 0.19) and mixed infection (0.20). No meaningful imbalance (standardized difference >0.3) was detected for any measured covariate. For readers who wish to see conventional *p*-values, these are provided in Supplementary Table S1. However, standardized differences (Table 1) are the primary balance metric as they are independent of sample size. All baseline and outcome variables were completely recorded for the 56 included patients; no missing data were present.

**Table 1:** Baseline characteristics of patients in the two groups.

Characteristic	AB-NPWT Group (*n* = 29)	Flap Group (*n* = 27)	Standardized Difference
Demographics			
Age (years), mean ± SD	65.4 ± 8.7	63.9 ± 9.2	0.17
Male sex, *n* (%)	17 (58.6)	15 (55.6)	0.06
Diabetes-related			
Diabetes duration (years), median (IQR)	12.0 (8.5–16.0)	10.0 (7.0–15.0)	0.21
HbA1c (%), mean ± SD	8.1 ± 1.5	7.9 ± 1.3	0.14
Neuropathy (10 g monofilament abnormal), *n* (%)	24 (82.8)	22 (81.5)	0.03
Bone health			
BMD T-score, mean ± SD	−3.0 ± 0.4	−2.9 ± 0.5	0.22
Ulcer characteristics			
Wagner grade 4, *n* (%)	11 (37.9)	11 (40.7)	0.06
Ulcer area (cm^2^), median (IQR)	8.5 (5.0–12.0)	9.0 (6.0–14.5)	0.16
Ulcer location, *n* (%)			
Forefoot	18 (62.1)	15 (55.6)	0.13
Midfoot	7 (24.1)	8 (29.6)	0.12
Hindfoot	4 (13.8)	4 (14.8)	0.03
Weight-bearing surface involved, *n* (%)	20 (69.0)	19 (70.4)	0.03
Vascular status			
ABI < 0.9, *n* (%)	9 (31.0)	7 (25.9)	0.11
Prior revascularization (history), *n* (%)	4 (13.8)	5 (18.5)	0.13
Renal function			
eGFR < 60 mL/min/1.73 m^2^, *n* (%)	8 (27.6)	6 (22.2)	0.12
Nutritional status			
Serum albumin (g/L), mean ± SD	34.2 ± 4.5	35.1 ± 4.8	0.19
Lifestyle			
Current smoker, *n* (%)	10 (34.5)	7 (25.9)	0.19
Comorbidities			
Hypertension, *n* (%)	21 (72.4)	19 (70.4)	0.04
Coronary artery disease, *n* (%)	9 (31.0)	8 (29.6)	0.03
Chronic kidney disease (stage 3–5), *n* (%)	6 (20.7)	5 (18.5)	0.06
Preoperative microbiology			
Single Gram-positive, *n* (%)	15 (51.7)	12 (44.4)	0.15
Mixed infection, *n* (%)	10 (34.5)	12 (44.4)	0.20
Other/No growth, *n* (%)	4 (13.8)	3 (11.1)	0.08

Data are presented as mean ± SD (normally distributed continuous), median (IQR) (non-normally distributed continuous), or *n* (%) (categorical). ABI, ankle-brachial index; BMD, bone mineral density; CAD, coronary artery disease; CKD, chronic kidney disease; eGFR, estimated glomerular filtration rate; IQR, interquartile range; SD, standard deviation. Standardized difference = (mean₁–mean₂)/pooled SD for continuous variables; for binary variables, difference in proportions divided by √[p(1-p)]. Values < 0.1 indicate good balance, 0.1–0.3 moderate imbalance, >0.3 meaningful imbalance. *P*-values are not shown in this table (see Supplementary Table S1 for *p*-values). No missing data for any variable in this table among the 56 included patients.

### Reconstructive subtypes

In the AB-NPWT pathway, the second-stage coverage methods were split-thickness skin grafting (19/29, 65.5%), direct closure (5/29, 17.2%), and small local flaps (5/29, 17.2%). In the flap-based pathway, flap types were local flaps (11/27, 40.7%), pedicled flaps (9/27, 33.3%), and free flaps (7/27, 25.9%). No significant differences in baseline characteristics were observed between flap subtypes (Table 2).

**Table 2:** Reconstructive subtypes within each treatment pathway.

Pathway/Subtype	*n* (%)	Operative time (min)	Blood loss (mL)*
AB-NPWT pathway (*n* = 29)			
Cement + split-thickness skin graft	19 (65.5)	78 ± 18	45 (25–70)
Cement + direct closure	5 (17.2)	65 ± 12	30 (20–50)
Cement + small local flap	5 (17.2)	110 ± 22	80 (60–110)
Flap-based pathway(*n* = 27)			
Local flap	11 (40.7)	145 ± 35	150 (120–200)
Pedicled flap	9 (33.3)	195 ± 40	220 (160–300)
Free flap	7 (25.9)	230 ± 38	300 (250–400)

Data are presented as *n* (%) for subtypes, mean ± SD for operative time (normally distributed), and median (IQR) for blood loss (non-normally distributed). AB-NPWT, antibiotic-loaded bone cement combined with negative pressure wound therapy; SD, standard deviation; IQR, interquartile range. Operative time and blood loss refer only to the Stage 2 definitive coverage procedure, not including Stage 1 debridement or additional procedures. Percentages are column percentages within each pathway.

### Perioperative parameters and primary outcomes

When considering only the definitive stage-2 procedure, AB-NPWT had significantly shorter operative time (85.6 vs. 186.3 min, *p* < 0.001) and lower blood loss (50 vs. 200 mL, *p* < 0.001) than flap transplantation. However, the AB-NPWT pathway required more total procedures (median 2 vs. 2, *p* = 0.023) and had a longer total hospital stay (28 vs. 22 days, *p* = 0.042) because of staged cement removal and secondary closure. Cumulative operative time (Stage 1 + Stage 2 + additional procedures) remained lower in the AB-NPWT group (135.8 vs. 230.1 min, *p* < 0.001), as did cumulative blood loss (75 vs. 220 mL, *p* < 0.001), reflecting the less invasive nature of cement placement and secondary closure compared to flap dissection (Table 3).

**Table 3:** Perioperative parameters, efficacy outcomes, and complications in the Two groups.

Parameter	AB-NPWT	Flap Group	*p*-value*
	(*n* = 29)	(*n* = 27)	
Perioperative parameters			
Stage 1 (debridement + NPWT)			
Operative time (min), mean ± SD	45.2 ± 12.5	43.8 ± 11.9	0.672
Intraoperative blood loss (mL), median (IQR)	20 (10–30)	20 (10–25)	0.891
Postoperative stay after Stage 1 (days), median (IQR)	7 (5–10)	6 (5–9)	0.342
Stage 2 (definitive coverage)			
Operative time (min), mean ± SD	85.6 ± 21.4	186.3 ± 45.8	<0.001
Intraoperative blood loss (mL), median (IQR)	50 (30–80)	200 (150–300)	<0.001
Postoperative stay after Stage 2 (days), median (IQR)	8 (6–12)	10 (7–14)	0.108
Cumulative (Stage 1 + Stage 2 + any additional)			
Total operative time (min), mean ± SD	135.8 ± 28.6	230.1 ± 52.3	<0.001
Total intraoperative blood loss (mL), median (IQR)	75 (45–110)	220 (170–320)	<0.001
Total hospital stay (days), median (IQR)	28 (19–38)	22 (16–30)	0.042
Number of surgical procedures per patient, median (IQR)	2 (2–3)	2 (2–2)	0.023
Efficacy and functional outcomes			
Complete wound healing rate, *n* (%)	25 (86.2)	24 (88.9)	0.754
Median healing time (days), M (IQR)	126 (98–167)	112 (84–151)	0.221
Limb salvage rate, *n* (%)	27 (93.1)	25 (92.6)	1.000
Ulcer recurrence rate, *n* (%)	4 (13.8)	3 (11.1)	1.000
AOFAS score at final follow-up, x¯ ± s	72.5 ± 10.3	75.8 ± 11.6	0.270
Overall complication rate, *n* (%)	7 (24.1)	9 (33.3)	0.446
Deep infection recurrence	4 (13.8)	4 (14.8)	
Material/flap-related	3 (10.3)	5 (18.5)	

Data are presented as mean ± SD (normally distributed continuous), median (IQR) (non-normally distributed continuous), or *n* (%) (categorical). AB-NPWT, antibiotic-loaded bone cement combined with negative pressure wound therapy; AOFAS, American orthopaedic foot & ankle society; M, median; IQR, interquartile range; SD, standard deviation; CI, confidence interval. Material-related complications include cement loosening and fracture. Flap-related complications include partial necrosis, vascular crisis, and donor site issues. In the AB-NPWT pathway, additional procedures (beyond Stage 1 and Stage 2) included cement removal (*n* = 29, 100%) and secondary closure (skin graft, direct closure, or small local flap) (*n* = 29, 100%). In the flap pathway, additional procedures included flap revision (*n* = 2, 7.4%) and debridement of partial necrosis (*n* = 1, 3.7%). No patient required more than 3 total surgical procedures.

* *P*-values are from independent t-tests (for normally distributed continuous variables), Mann–Whitney U tests (for non-normally distributed continuous variables), or chi-square/Fisher's exact tests (for categorical variables), as appropriate. The AOFAS score comparison was adjusted for follow-up duration using linear regression; the unadjusted *p*-value shown is from an independent *t*-test. The adjusted mean difference was −2.4 (95% CI −7.1 to 2.3, *p* = 0.316).

**Figure 5:**
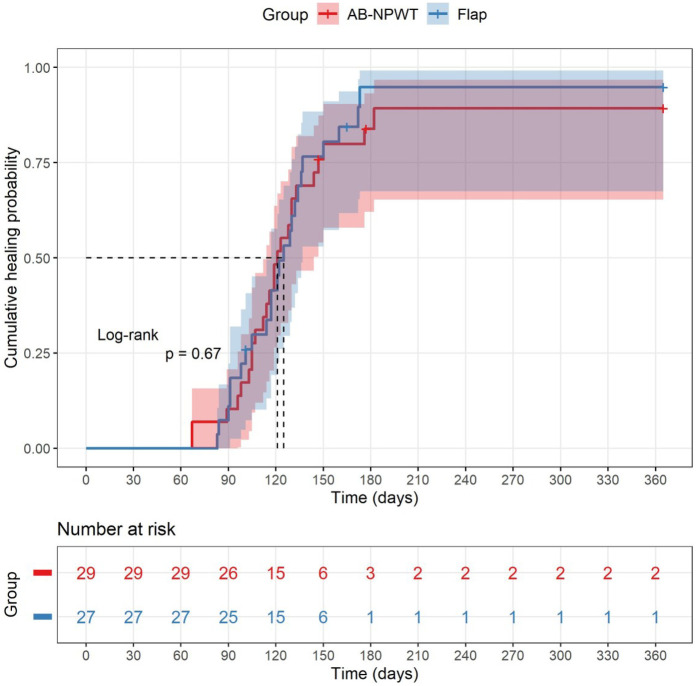
Kaplan–Meier curves for time to complete wound healing in the AB-NPWT and flap groups. Censoring points indicate amputation or end of follow-up. Log-rank *p* = 0.221.

At 12 months, complete wound healing was achieved in 25/29 (86.2%) patients in the AB-NPWT group and 24/27 (88.9%) in the flap group (*p* = 0.754). Limb salvage rates were 93.1% (27/29) and 92.6% (25/27), respectively (*p* = 1.000). By Kaplan–Meier analysis, the estimated median healing time was 126 days (95% CI 105–147) in the AB-NPWT group and 112 days (95% CI 93–131) in the flap group. The log-rank test showed no significant difference between groups (*χ*^2^ = 1.50, *p* = 0.221). The unadjusted hazard ratio for healing (AB-NPWT vs. flap) was 0.85 (95% CI 0.58–1.24). After adjustment for the eight pre-specified covariates (age, Wagner grade, ulcer area, PAD, smoking, HbA1c, exposed bone/tendon, and ASA score), the adjusted HR for healing was 0.87 (95% CI 0.60–1.26, *p* = 0.456). The cumulative healing probabilities at 12 months were 86.2% (95% CI 72.5–93.5) in the AB-NPWT group and 88.9% (95% CI 76.0–95.1) in the flap group (Figure 5).

### Crossover sensitivity analysis

Sensitivity analysis for crossover patients—In the primary intention-to-treat analysis (including all 29 AB-NPWT patients as originally assigned), the unadjusted OR for complete healing was 0.78 (95% CI 0.33–1.85) and for limb salvage was 1.08 (95% CI 0.37–3.15).

After excluding the 5 crossover patients (AB-NPWT *n* = 24), the healing rate was 20/24 (83.3%) vs. 24/27 (88.9%) in the flap group (OR 0.63, 95% CI 0.24–1.64, *p* = 0.342). Limb salvage was 23/24 (95.8%) vs. 25/27 (92.6%) (OR 1.77, 95% CI 0.32–9.82, *p* = 0.508). Median healing time (Kaplan–Meier) was 130 days (95% CI 108–152) in the AB-NPWT group vs. 112 days (95% CI 93–131) in the flap group (log-rank *p* = 0.285).

In the as-treated analysis (crossover patients reclassified into flap group; AB-NPWT *n* = 24, flap *n* = 32), the healing rate was 20/24 (83.3%) vs. 29/32 (90.6%) (OR 0.55, 95% CI 0.22–1.38, *p* = 0.202). Limb salvage was 23/24 (95.8%) vs. 30/32 (93.8%) (OR 1.47, 95% CI 0.29–7.45, *p* = 0.644). Median healing time was 130 days vs. 114 days (log-rank *p* = 0.312).

In both sensitivity analyses, the direction and magnitude of effect estimates were consistent with the primary ITT analysis (all confidence intervals crossed 1 for OR and HR, and *p* > 0.05). No statistically significant differences emerged between groups. These analyses indicate that crossover did not materially bias the primary findings.

### Complications and functional outcomes

Overall complication rates were similar between groups (24.1% vs. 33.3%, *p* = 0.446). However, complication profiles differed: the AB-NPWT group experienced more cement-related events (loosening/fracture, 10.3%) and deep infection recurrence (13.8%), while the flap group had more flap-related complications (partial necrosis/donor site issues, 18.5%) and similar infection recurrence (14.8%). One systemic complication occurred in each group (pneumonia). Ulcer recurrence within 12 months of complete wound healing occurred in 4 patients (13.8%) in the AB-NPWT group and 3 patients (11.1%) in the flap group (*p* = 0.754).

### Functional outcomes (AOFAS)

The median follow-up duration for AOFAS assessment was 15.5 months (IQR 12–20, range 12–26) in the AB-NPWT group and 16.0 months (IQR 13–22, range 12–24) in the flap group (*p* = 0.612). In unadjusted analysis, mean AOFAS scores were 72.5 ± 10.3 in the AB-NPWT group vs. 75.8 ± 11.6 in the flap group (*p* = 0.270).

To account for variable follow-up timing, we performed a sensitivity analysis including only patients with AOFAS measured at 12 months (±1 month): 18 patients in the AB-NPWT group (62.1%) and 16 patients in the flap group (59.3%). In this time-standardized subset, mean AOFAS scores were 71.8 ± 9.9 vs. 74.2 ± 10.5 (*p* = 0.489).

In a linear regression model adjusting for follow-up duration (months) as a continuous covariate, the estimated between-group difference in AOFAS score was −2.4 points (95% CI −7.1 to 2.3, *p* = 0.316) in favor of the flap group, which was not statistically significant. Follow-up duration itself was not associated with AOFAS score (*β* = 0.12 points per month, 95% CI −0.22 to 0.46, *p* = 0.482). These analyses confirm that the lack of significant difference is not explained by variable follow-up timing.

### Subgroup analysis by ulcer severity

An exploratory descriptive subgroup analysis by Wagner grade was performed (Table 4). In Wagner grade 3 ulcers, healing rates were 94.4% (17/18) in the AB-NPWT group and 93.8% (15/16) in the flap group; limb salvage was 100% in both groups. In Wagner grade 4 ulcers, healing rates were 72.7% (8/11) and 81.8% (9/11), respectively; limb salvage was 81.8% in both groups. Median healing times were longer in grade 4 ulcers (158 and 145 days) than in grade 3 ulcers (105 and 98 days). These findings are descriptive and hypothesis-generating; no statistical comparisons were performed.

**Table 4:** Exploratory subgroup analysis by Wagner grade (descriptive only).

Parameter	Wagner Grade 3 Patients		Wagner Grade 4 Patients	
Group	Cement Group	Flap Group	Cement Group	Flap Group
	(*n* = 18)	(*n* = 16)	(*n* = 11)	(*n* = 11)
Healing rate, *n* (%)	17 (94.4)	15 (93.8)	8 (72.7)	9 (81.8)
Limb salvage rate, *n* (%)	18 (100)	16 (100)	9 (81.8)	9 (81.8)
Median healing time (days), M (IQR)	105 (80–140)	98 (75–132)	158 (130–195)	145 (120–180)

This subgroup analysis is exploratory and descriptive. No statistical comparisons (*p*-values, confidence intervals, or interaction tests) were performed due to limited sample size. Data are presented as *n* (%) for healing and limb salvage rates, and as median (IQR) for healing time. IQR, interquartile range. Percentages are column percentages within each Wagner grade and treatment group. Findings should be interpreted as hypothesis-generating only.

### Treatment pathway differences

In the AB-NPWT group, second-stage coverage methods included split-thickness skin grafting (65.5%, 19/29), direct closure (17.2%, 5/29), and small local flaps (17.2%, 5/29). Thus, 17.2% of patients initially allocated to cement ultimately received flap surgery, though these were less invasive local flaps.

### Adjusted analyses

Adjusted analyses were performed using the eight pre-specified covariates (age, Wagner grade, ulcer area, PAD, smoking, HbA1c, exposed bone/tendon, ASA score). The results remained consistent with the unadjusted findings. In multivariable logistic regression, the adjusted odds ratio for complete wound healing in the AB-NPWT group compared to the flap group was 0.79 (95% CI 0.31–2.01, *p* = 0.624). For limb salvage, the adjusted odds ratio was 1.08 (95% CI 0.42–2.77, *p* = 0.865). In Cox regression for healing time, the adjusted hazard ratio was 0.87 (95% CI 0.60–1.26, *p* = 0.456).

Propensity score matching yielded 23 well-balanced pairs (standardized differences < 0.1 for all covariates). In the matched sample, healing rates were 87.0% (20/23) in the AB-NPWT group vs. 91.3% (21/23) in the flap group (*p* = 0.635); limb salvage rates were 95.7% (22/23) in both groups (*p* = 1.000). Inverse probability of treatment weighting (IPTW) produced similar effect estimates: for complete wound healing, OR = 0.81 (95% CI 0.35–1.88); for limb salvage, OR = 1.05 (95% CI 0.44–2.51); for healing time, HR = 0.88 (95% CI 0.63–1.23).

Detailed results of the IPTW analysis are provided in Supplementary Table S2. Covariate balance before and after propensity score matching is shown in Supplementary Table S3. Cox model diagnostics, including the Schoenfeld residual test for the proportional-hazards assumption (global *p* = 0.836), are presented in the Supplementary Appendix (Cox Model Diagnostics section). These adjusted analyses confirm that the absence of statistically significant between-group differences is not explained by measured confounding (Table 5).

**Table 5:** Adjusted effect estimates for primary outcomes.

Outcome	Model	Effect estimate (AB-NPWT vs. Flap)	95% CI	*p*-value
Complete wound healing	Unadjusted OR	0.78	0.33–1.85	0.754
	Adjusted OR*	0.79	0.31–2.01	0.624
	Propensity-matched OR	0.65	0.21–2.01	0.635
Limb salvage	Unadjusted OR	1.08	0.37–3.15	1.000
	Adjusted OR	1.08	0.42–2.77	0.865
	Propensity-matched OR	1.00	0.23–4.35	1.000
Healing time	Unadjusted HR	0.85	0.58–1.24	0.221
	Adjusted HR†	0.87	0.60–1.26	0.456

OR, odds ratio; HR, hazard ratio; CI, confidence interval; AB-NPWT, antibiotic-loaded bone cement combined with negative pressure wound therapy; HbA1c, glycated haemoglobin; PAD, peripheral arterial disease (ankle-brachial index < 0.9). Adjusted OR and adjusted HR are from multivariable models including the following eight pre-specified covariates: age, Wagner grade (3 vs. 4), ulcer area (cm²), peripheral arterial disease (ABI < 0.9), smoking status, baseline HbA1c, exposed bone or tendon (yes/no), and ASA physical status (I–III). Propensity score matching was performed using the same eight covariates. Unadjusted ORs and HRs are from models without covariates. All confidence intervals are 95%.

## Discussion

To our knowledge, direct comparative data between AB-NPWT and flap transplantation in T2DM-OP patients are scarce. This retrospective cohort study provides preliminary observational evidence comparing these two reconstructive pathways in this specific high-risk population. Within the limitations of our retrospective design and small sample size, the absence of statistically significant differences in primary endpoints may suggest that the two pathways achieve similar ultimate outcomes despite different mechanisms. However, this finding should be considered exploratory and hypothesis-generating rather than definitive. A lack of statistical significance should not be interpreted as evidence of equivalence. The confidence intervals for effect estimates were wide (e.g., OR for healing 0.34–1.98), meaning that clinically important benefits of either strategy cannot be ruled out.

First, diabetic microangiopathy and neuropathy may attenuate the vascular advantage conferred by flaps, limiting their theoretical superiority (18). Second, osteoporosis compromises the mechanical stability of flap fixation on the recipient bone surface, potentially increasing the risk of micro-motion and graft failure (19, 20). Conversely, AB-NPWT provides a different healing pathway: local antibiotic elution eradicates infection, while the cement spacer maintains dead space and skeletal alignment, allowing secondary intention healing or subsequent less-invasive coverage. This “infection control + mechanical support” mechanism may be particularly effective when vascularity is impaired and bone quality is poor.

AB-NPWT demonstrated clear benefits in operative time and blood loss, reflecting its less invasive nature. However, this came at the expense of a trend toward longer total healing time (median 126 vs. 112 days, not statistically significant) and the requirement for a second procedure in all patients. Thus, the choice between strategies involves a trade-off: AB-NPWT offers a gentler initial surgery but prolongs the overall treatment course, whereas flap transplantation achieves one-stage coverage at the cost of greater surgical trauma (21–23). Clinicians must weigh these factors against patient preferences, comorbidities, and healthcare resources.

The differing complication patterns align with the mechanistic basis of each approach. Cement-related complications (loosening, fracture) arise from the biomechanical mismatch between rigid cement and osteoporotic bone, while infection recurrence reflects incomplete bacterial eradication. Flap complications, including partial necrosis and donor site morbidity, stem from the technical demands of microsurgery and the patient' s impaired healing capacity. Notably, the similar overall complication rates suggest that both strategies carry inherent risks that must be carefully managed.

An intriguing finding is that 17.2% of patients in the cement group ultimately received small local flaps during Stage 2. This suggests that AB-NPWT can serve as a “bridge” to definitive coverage: it first achieves infection control and wound stabilization, after which a less extensive flap becomes feasible (14, 24). This sequential approach may expand treatment options for patients initially unsuitable for complex reconstruction. Future studies should identify predictors of successful bridging and evaluate long-term outcomes.

Our findings should be interpreted within the framework of the 2023 IWGDF/IDSA guidelines, which emphasize multidisciplinary care including off-loading, revascularization, and metabolic control (7–10). The similar outcomes observed between the two reconstructive pathways suggest that, when guideline-recommended adjunctive care is provided, the choice of definitive coverage method may be less critical than ensuring adequate infection control and patient selection. However, this hypothesis requires prospective testing.

In an exploratory descriptive subgroup analysis, patients with Wagner grade 4 ulcers had numerically lower healing and limb salvage rates (approximately 80%) compared to those with grade 3 ulcers (near 100%). This observation, while not statistically tested due to limited sample size, suggests that ulcer depth and bone involvement remain important prognostic factors (25). In this severe subgroup, both reconstructive pathways appeared challenged, and patients should be counseled about the heightened risk of treatment failure and amputation. These findings are hypothesis-generating and require confirmation in larger studies.

Our adjusted analyses accounted for measured confounders including age, disease severity (Wagner grade, ulcer area, exposed bone/tendon), vascular status (PAD), metabolic control (HbA1c), lifestyle (smoking), and global frailty (ASA score). However, several factors that influenced treatment selection in clinical practice—such as donor-site feasibility, availability of microsurgical expertise, and patient preference—were not recorded in a standardised quantitative format and therefore could not be adjusted for. Residual confounding from these unmeasured variables remains possible.

This study has several limitations. First, the retrospective, single-center design introduces potential selection bias and limits generalizability. Second, treatment allocation was non-randomized and determined by the attending surgeon, creating a high risk of confounding by indication despite our adjusted analyses. Third, the moderate sample size (*n* = 56) resulted in wide confidence intervals (e.g., OR for healing 0.34–1.98), meaning that clinically important differences cannot be ruled out. Fourth, the 12-month follow-up may be insufficient to capture late recurrences or late complications, in the time-to-healing analysis, amputation was treated as censoring rather than a competing event; this is a simplification that we acknowledge. Fifth, crossover occurred in 17.2% of the AB-NPWT group; although sensitivity analyses did not change the conclusions, residual bias cannot be excluded. Sixth, the lack of blinding of outcome assessors may have introduced detection bias. Seventh, although we performed multivariable and propensity score adjustments, residual confounding from unmeasured variables (e.g., detailed nutritional status, frailty, surgeon expertise) remains possible. Eighth, the heterogeneity within each treatment pathway (different flap subtypes, secondary closure methods) limits precision; we lacked power for subtype-specific analyses. Ninth, the diagnostic criteria for infection control and osteomyelitis, while standardized, still involved some clinical judgment. Tenth, the AOFAS functional scores were measured at variable follow-up times (12–26 months); although we adjusted for follow-up duration, residual confounding remains. Eleventh, we did not perform a formal cost-effectiveness analysis. Twelfth, the findings are from a single center in China and may not generalize to other healthcare systems. Thirteenth, the absence of statistically significant differences should not be interpreted as evidence of equivalence; the study was not powered for non-inferiority testing. These limitations underscore the exploratory nature of our findings. Furthermore, several treatment-selection variables (donor-site feasibility, microsurgical resource availability, detailed patient preference) were not recorded in a standardised quantitative manner and could not be adjusted for, leaving potential for residual confounding.

In our practice, patients with larger defects, exposed bone, or good functional reserve were preferentially assigned to the flap pathway, while frailer patients with marginal perfusion or smaller defects received AB-NPWT. The non-randomised, surgeon-driven allocation is the principal threat to validity. The treatment-selection algorithm (Figure 4) shows that multiple factors guided pathway assignment. Patients with larger defects, exposed bone/tendon, and good functional reserve were preferentially assigned to flap surgery, while frailer patients (higher ASA score) and those with marginal perfusion or smaller defects received AB-NPWT. This creates complex, bidirectional confounding by indication. It is not possible to assume a single direction of residual bias; unmeasured factors (e.g., patient preference, social support, subtle differences in wound care) may also affect outcomes. Although we expanded our adjustment set to include exposed bone and ASA score, and performed propensity score matching, residual confounding cannot be excluded. Therefore, our findings should be interpreted as exploratory and hypothesis-generating, not causal. Despite this, we observed no statistically significant differences, suggesting that any true advantage of flaps may be attenuated by this bias. Conversely, the crossover of 5 AB-NPWT patients to local flaps could have improved outcomes in the AB-NPWT group, biasing results toward the null. Our sensitivity analyses excluding these patients did not change the conclusions, but residual bias cannot be excluded. Unmeasured confounders (e.g., frailty, social support, adherence to off-loading, subtle differences in wound care) may also affect outcomes. Therefore, our findings should be interpreted as exploratory and hypothesis-generating, not causal.

## Conclusions

In this retrospective cohort, AB-NPWT and flap transplantation were associated with similar observed outcomes in wound healing, limb salvage, and functional recovery. AB-NPWT was associated with reduced surgical trauma but may involve a longer total treatment course, whereas flap transplantation achieved definitive coverage in a single operative session after debridement. These findings are exploratory and hypothesis-generating; they should not be interpreted as evidence of equivalence. Treatment selection should be individualized based on wound characteristics, patient systemic status, and healthcare resources. Future studies should be multicentre and prospectively designed, employ pre-specified pathway selection criteria, capture a broader range of confounders (including frailty and detailed wound characteristics), and include longer follow-up (≥24 months) to assess late recurrence and sustained functional outcomes.

## Data Availability

The raw data supporting the conclusions of this article will be made available by the authors, without undue reservation.
