# Supplementary material for: Staged antibiotic-loaded cement-based reconstruction versus flap-based reconstruction for complex diabetic foot defects in patients with osteoporosis: a retrospective cohort study
Source: Front Surg. 2026 May 20;13:1834390. doi: 10.3389/fsurg.2026.1834390 (PMC13230152; doi:10.3389/fsurg.2026.1834390)
Supplement: Supplementary file 1 [file Table1.docx]

**Table S1. Baseline Characteristics with p-values**

| Characteristic | AB-NPWT Group (n=29) | Flap Group (n=27) | p-value* |
| --- | --- | --- | --- |
| **Demographics** |  |  |  |
| Age (years), mean ± SD | 65.4 ± 8.7 | 63.9 ± 9.2 | 0.530 |
| Male sex, n (%) | 17 (58.6) | 15 (55.6) | 0.830 |
| **Diabetes-related** |  |  |  |
| Diabetes duration (years), median (IQR) | 12.0 (8.5–16.0) | 10.0 (7.0–15.0) | 0.357 |
| HbA1c (%), mean ± SD | 8.1 ± 1.5 | 7.9 ± 1.3 | 0.594 |
| Neuropathy (10g monofilament abnormal), n (%) | 24 (82.8) | 22 (81.5) | 0.999 |
| **Bone health** |  |  |  |
| BMD T-score, mean ± SD | -3.0 ± 0.4 | -2.9 ± 0.5 | 0.422 |
| **Ulcer characteristics** |  |  |  |
| Wagner grade 4, n (%) | 11 (37.9) | 11 (40.7) | 0.916 |
| Ulcer area (cm²), median (IQR) | 8.5 (5.0–12.0) | 9.0 (6.0–14.5) | 0.463 |
| Ulcer location, n (%) |  |  | 0.872† |
| – Forefoot | 18 (62.1) | 15 (55.6) |  |
| – Midfoot | 7 (24.1) | 8 (29.6) |  |
| – Hindfoot | 4 (13.8) | 4 (14.8) |  |
| Weight-bearing surface involved, n (%) | 20 (69.0) | 19 (70.4) | 0.999 |
| **Vascular status** |  |  |  |
| ABI <0.9, n (%) | 9 (31.0) | 7 (25.9) | 0.766 |
| Prior revascularization (history), n (%) | 4 (13.8) | 5 (18.5) | 0.727 |
| **Renal function** |  |  |  |
| eGFR <60 mL/min/1.73m², n (%) | 8 (27.6) | 6 (22.2) | 0.759 |
| **Nutritional status** |  |  |  |
| Serum albumin (g/L), mean ± SD | 34.2 ± 4.5 | 35.1 ± 4.8 | 0.470 |
| **Lifestyle** |  |  |  |
| Current smoker, n (%) | 10 (34.5) | 7 (25.9) | 0.566 |
| **Comorbidities** |  |  |  |
| Hypertension, n (%) | 21 (72.4) | 19 (70.4) | 0.999 |
| Coronary artery disease, n (%) | 9 (31.0) | 8 (29.6) | 0.999 |
| Chronic kidney disease (stage 3–5), n (%) | 6 (20.7) | 5 (18.5) | 0.999 |
| **Preoperative microbiology** |  |  |  |
| Single Gram-positive, n (%) | 15 (51.7) | 12 (44.4) | 0.779† |
| Mixed infection, n (%) | 10 (34.5) | 12 (44.4) |  |
| Other/No growth, n (%) | 4 (13.8) | 3 (11.1) |  |

*P-values from independent t-test (continuous, normal), Mann-Whitney U test (continuous, non-normal), chi-square test (categorical), or Fisher’s exact test (categorical with small cell sizes), as appropriate. †P-value for overall distribution (chi-square test).

**Abbreviations:** ABI, ankle-brachial index; BMD, bone mineral density; eGFR, estimated glomerular filtration rate; IQR, interquartile range; SD, standard deviation.

Note: Standardized differences (presented in Table 1) are the primary balance metric for this observational study, as they are independent of sample size. P-values are provided below for readers who wish to see conventional significance testing, but they should not be interpreted as evidence of balance or imbalance due to their dependence on sample size.

**Table S2. Inverse Probability of Treatment Weighting (IPTW) Estimates**

| Outcome | Effect estimate (AB‑NPWT vs. Flap) | 95% CI | p‑value |
| --- | --- | --- | --- |
| Complete wound healing (OR) | 0.81 | 0.35–1.88 | 0.621 |
| Limb salvage (OR) | 1.05 | 0.44–2.51 | 0.912 |
| Healing time (HR) | 0.88 | 0.63–1.23 | 0.458 |

**Note:** IPTW was performed using the same covariate set as the primary adjusted analysis (age, Wagner grade, ulcer area, PAD, smoking, HbA1c, exposed bone/tendon, ASA score). Weights were stabilised and truncated at the 1st and 99th percentiles. OR: odds ratio; HR: hazard ratio; CI: confidence interval.

**Table S3. Balance of Covariates Before and After Propensity Score Matching**

| Covariate | Before matching | After matching (23 pairs) |
| --- | --- | --- |
|  | Standardized difference | Standardized difference |
| Age | 0.17 | 0.04 |
| Wagner grade (4 vs. 3) | 0.06 | 0.02 |
| Ulcer area (cm²) | 0.16 | 0.05 |
| PAD (ABI <0.9) | 0.11 | 0.03 |
| Smoking | 0.19 | 0.06 |
| HbA1c (%) | 0.14 | 0.04 |
| Exposed bone/tendon | 0.24 | 0.07 |
| ASA score (I–III) | 0.21 | 0.05 |

**Note:** Standardized differences <0.1 indicate good balance. PAD: peripheral arterial disease; ABI: ankle‑brachial index; HbA1c: glycated haemoglobin; ASA: American Society of Anesthesiologists.

**Cox Model Diagnostics – Proportional Hazards Assumption**

The proportional‑hazards assumption for the Cox regression model of healing time was tested using Schoenfeld residuals. The global test yielded χ² = 4.23, df = 8, p = 0.836, indicating no violation of the proportional‑hazards assumption. Individual covariate tests also showed no significant deviations (all p > 0.05). Therefore, the Cox model was appropriate for the time‑to‑healing analysis.
